# WIse CHoices in the ICU (WICH‐ICU): Protocol and Statistical Analysis Plan for a Stepped‐Wedge Cluster Randomized Trial of Choosing Wisely Interventions in Swedish Intensive Care Units

**DOI:** 10.1111/aas.70339

**Published:** 2026-09-23

**Authors:** Emma Larsson, Arman Valadkhani, Jesper Eriksson, Erik Svensk, Fredrik Hammarskjöld, Markus Castegren, Peter Bentzer, Nicklas Nielsen, Kristina Svennerholm, Mikael Eriksson, Max Bell

**Affiliations:** ^1^ Department of Physiology and Pharmacology Karolinska Institutet Stockholm Sweden; ^2^ Department of Perioperative Medicine and Intensive Care (PMI) Karolinska University Hospital Stockholm Sweden; ^3^ Department of Anesthesiology and Intensive Care Sundsvall Hospital Sundsvall Sweden; ^4^ Department of Biomedical and Clinical Sciences Linköping University Linköping Sweden; ^5^ Department of Anaesthesia and Intensive Care Medicine Ryhov County Hospital Jönköping Sweden; ^6^ CLINTEC, Karolinska Institutet Stockholm Sweden; ^7^ Centre for Clinical Research Sörmland, Uppsala University Uppsala Sweden; ^8^ Anaesthesiology and Intensive Care, Department of Clinical Sciences, Malmö Lund University Lund Sweden; ^9^ Department of Clinical Sciences, Anaesthesia and Intensive Care Lund University, Helsingborg Hospital Helsingborg Sweden; ^10^ Department of Anaesthesiology and Intensive Care Institute of Clinical Sciences, Sahlgrenska Academy, University of Gothenburg Gothenburg Sweden; ^11^ Department of Anaesthesiology and Intensive Care Sahlgrenska University Hospital Gothenburg Region Västra Götaland Sweden; ^12^ Department of Surgical Sciences Anaesthesia and Intensive Care, Uppsala University Uppsala Sweden; ^13^ Department of Anaesthesia and Intensive Care Uppsala University Hospital Uppsala Sweden

**Keywords:** Choosing Wisely, cluster randomized trial, de‐implementation, healthcare overuse, intensive care, stepped‐wedge

## Abstract

**Background:**

Healthcare overuse, including unnecessary diagnostic testing and procedures, contributes to patient harm, increased healthcare costs, and resource waste. The Choosing Wisely initiative aims to reduce low‐value care through evidence‐based recommendations. However, limited evidence exists regarding the safety and effectiveness of implementing Choosing Wisely recommendations in intensive care settings.

**Methods:**

The WICH‐ICU is a registry‐based stepped‐wedge cluster randomized controlled trial designed to evaluate the implementation of specific Choosing Wisely interventions in Swedish intensive care units (ICUs). The study will include adult patients treated in participating Swedish ICUs. The intervention consists of three parts of the Choosing Wisely recommendations: (1) reduced frequency of arterial blood gas sampling, (2) reduced routine laboratory testing, and (3) reduced routine chest radiography. The primary outcome is 30‐day mortality. Secondary outcomes include ICU length of stay, duration of mechanical ventilation (invasive and noninvasive), continuous renal replacement therapy duration, and ICU readmission within 72 h. Intervention data, testing intensity (arterial blood gas samples, laboratory tests, and chest radiographs per patient‐day), will be collected through local monitoring, while outcome data will be obtained from the Swedish Intensive Care Registry (SIR). The stepped‐wedge design allows each participating ICU to serve as its own control, with randomized timing of intervention implementation.

**Discussion:**

This study will provide evidence on the safety and effectiveness of implementing Choosing Wisely recommendations in intensive care settings. The stepped‐wedge cluster randomized design minimizes contamination while allowing all participating sites to eventually receive the intervention. The results will inform evidence‐based de‐implementation strategies in critical care.

**Trial Registration:**

ClinicalTrials.gov identifier: NCT07013175

## 1 Background

Healthcare systems worldwide face mounting pressure to optimize resource utilization while maintaining high‐quality patient care. Healthcare overuse—defined as the provision of medical services when the potential for harm exceeds the potential for benefit—represents a significant challenge in modern medicine [1]. This phenomenon is particularly pronounced in intensive care units (ICUs), where the critical nature of patient conditions often drives defensive medicine practices and the routine ordering of diagnostic tests and procedures [2, 3].

The Choosing Wisely (CW) initiative, launched in 2012 by the American Board of Internal Medicine Foundation, represents a physician‐led effort to advance conversations between clinicians and patients about avoiding unnecessary medical tests, treatments, and procedures [4, 5]. The initiative has since expanded globally, with professional societies developing evidence‐based recommendations to reduce low‐value care [6]. However, despite widespread adoption of CW recommendations, implementation in clinical practice remains challenging, particularly in high‐acuity settings such as ICUs [7].

### 1.1 Rationale for CW in Intensive Care

ICUs are characterized by high resource utilization, complex patient populations, and frequent diagnostic testing [8, 9]. Several factors contribute to potential overuse in ICU settings:

1. *Defensive medicine practices*: The critical nature of ICU patients often leads to overcautious approaches, including frequent laboratory testing and imaging studies [10].
2. *Routine ordering packages*: Many ICU protocols include standing orders for daily laboratory tests and imaging studies, regardless of clinical indications [11].
3. *Shift‐based care*: The 24‐h nature of ICU care, with multiple handoffs between providers, may contribute to redundant testing [12].
4. *Technological availability*: The immediate availability of diagnostic technologies in ICU settings may lower the threshold for ordering tests [13].

### 1.2 Evidence for CW Interventions in Critical Care

Several CW recommendations specifically target intensive care practices:

*Arterial blood gas (ABG) sampling*: Routine ABG sampling in stable ICU patients may not improve clinical outcomes while contributing to iatrogenic anemia through cumulative diagnostic blood loss, and increasing healthcare resource use and the risk of complications; in patients without indwelling arterial access it also causes avoidable discomfort [14]. Evidence suggests that stable patients on mechanical ventilation may not require hourly ABG sampling, with extended intervals being safe and cost‐effective [15].

*Laboratory testing*: Daily routine laboratory testing in stable ICU patients has been questioned, with studies suggesting that selective testing based on clinical indication may be equally safe while reducing costs and iatrogenic anemia [16, 17]. The Society of Critical Care Medicine has specifically recommended against daily laboratory testing in stable ICU patients [18].

*Chest radiography*: Daily routine chest x‐rays in ICU patients, particularly those on mechanical ventilation, have limited diagnostic yield and may not improve clinical outcomes [19, 20]. Multiple professional societies recommend against routine daily chest radiography in favor of clinically indicated imaging [18, 21].

### 1.3 Knowledge Gaps and Study Rationale

Despite theoretical benefits and professional society recommendations, several knowledge gaps exist regarding CW implementation in intensive care:

1. *Safety concerns*: Limited high‐quality evidence exists regarding the safety of reducing diagnostic testing frequency in ICU patients [22].
2. *Implementation strategies*: Effective methods for implementing CW recommendations in ICU settings remain poorly understood [23].
3. *Patient outcomes*: The impact of CW interventions on patient‐centered outcomes in critical care requires further investigation [24].
4. *Healthcare utilization*: The broader effects of CW implementation on ICU resource utilization and efficiency need evaluation [25].
5. *Environmental impact*: The environmental impact of CW interventions in an ICU setting is unclear [26].

### 1.4 Swedish Healthcare Context

Sweden's tax‐funded public healthcare system, characterized by universal coverage and integrated delivery systems, provides an ideal setting for evaluating the systematic implementation of CW interventions. The Swedish Intensive Care Registry (SIR), established in 2001, captures comprehensive data on all ICU admissions nationwide, enabling robust outcome assessment [27]. The registry's high coverage rate (> 95% of Swedish ICU beds) and standardized data collection procedures facilitate large‐scale implementation research [28, 29].

Swedish ICU practice patterns align with international standards, with similar approaches to diagnostic testing and monitoring. However, regional variations exist in practice, providing natural variation for studying implementation effects [30, 31].

## 2 Objectives

### 2.1 Primary Objective

To evaluate the safety and effectiveness of implementing specific CW interventions in Swedish ICUs by assessing whether they are non‐inferior to usual care with regards to 30‐day mortality.

### 2.2 Secondary Objectives

1. To assess the impact of CW interventions on ICU length of stay.
2. To evaluate effects on invasive mechanical ventilation duration.
3. To evaluate effects on noninvasive mechanical ventilation duration.
4. To evaluate effects on continuous renal replacement therapy duration.
5. To evaluate effects on unplanned readmission.
6. To analyze changes in diagnostic testing patterns and healthcare resource utilization.
7. To analyze environmental impact.

### 2.3 Hypotheses

We hypothesize that the implementation of targeted CW interventions in Swedish ICUs will:

1. Reduce diagnostic testing frequency without compromising patient safety.
2. Maintain non‐inferior clinical outcomes while improving resource efficiency.
3. Be feasible to implement across diverse ICU settings.
4. Reduce resource use and environmental impact without negatively impacting quality indicators, such as ICU length of stay and unplanned ICU readmission within 72 h.

### 2.4 Trial Registration

ClinicalTrials.gov identifier: NCT07013175 (first posted on June 17, 2025; last updated on November 21, 2025).

### 2.5 Trial Status

Protocol version [2.0], dated August 8, 2026; version 1.0 dated November 24, 2025.

Recruitment opened on November 3, 2025 and is scheduled to close on November 30, 2026. The trial is registered at ClinicalTrials.gov (NCT07013175, https://clinicaltrials.gov/study/NCT07013175), first posted on June 17, 2025. This publication constitutes both the trial protocol and the statistical analysis plan; the full versions of both documents are deposited with the trial registration record and are available from the corresponding author on request. Results will be reported in a peer‐reviewed publication regardless of the direction of the findings, posted to the trial registry, and communicated to the participating ICUs and the wider Swedish intensive care community through SIR and SFAI. Authorship of trial publications follows ICMJE criteria; no professional medical writers were engaged. Cluster randomization and the design described here were finalized before recruitment opened; version 1.0 of the written protocol was filed on November 24, 2025. Version 2.0 differed from version 1.0 in the addition of a prespecified fidelity threshold for interpreting the primary result, a quantitative trigger for safety review, and expanded reporting of trial oversight and open‐science provisions. Version 3.0, prepared in response to peer review, adds a prespecified analysis model for the testing‐intensity outcomes, a per‐protocol analysis of the primary outcome reported alongside the intention‐to‐treat analysis, a hierarchical rule subordinating testing‐intensity and environmental outcomes to the primary safety outcome, and a contemporaneous comparison with nonparticipating SIR units. In no version have changes been made to eligibility, the intervention, the primary outcome, the non‐inferiority margin, or the primary analysis model. All versions were finalized before any outcome data were extracted or examined.

One participating unit, a small cardiothoracic ICU, withdrew from the trial on November 5, 2025, after cluster randomization and before the first clusters transitioned into intervention. Thirty‐seven units comprising 36 clusters therefore contributed data. The randomization sequence and the power calculation reported below are those prespecified for the 38 units (37 clusters) randomized; the withdrawal reduces the projected number of care episodes by approximately 197 (0.9%), so no recalculated power analysis will be done. The withdrawal and its timing will be shown in the CONSORT flow diagram of the primary‐results publication.

## 3 Methods

### 3.1 Study Design

**FIGURE 1:**
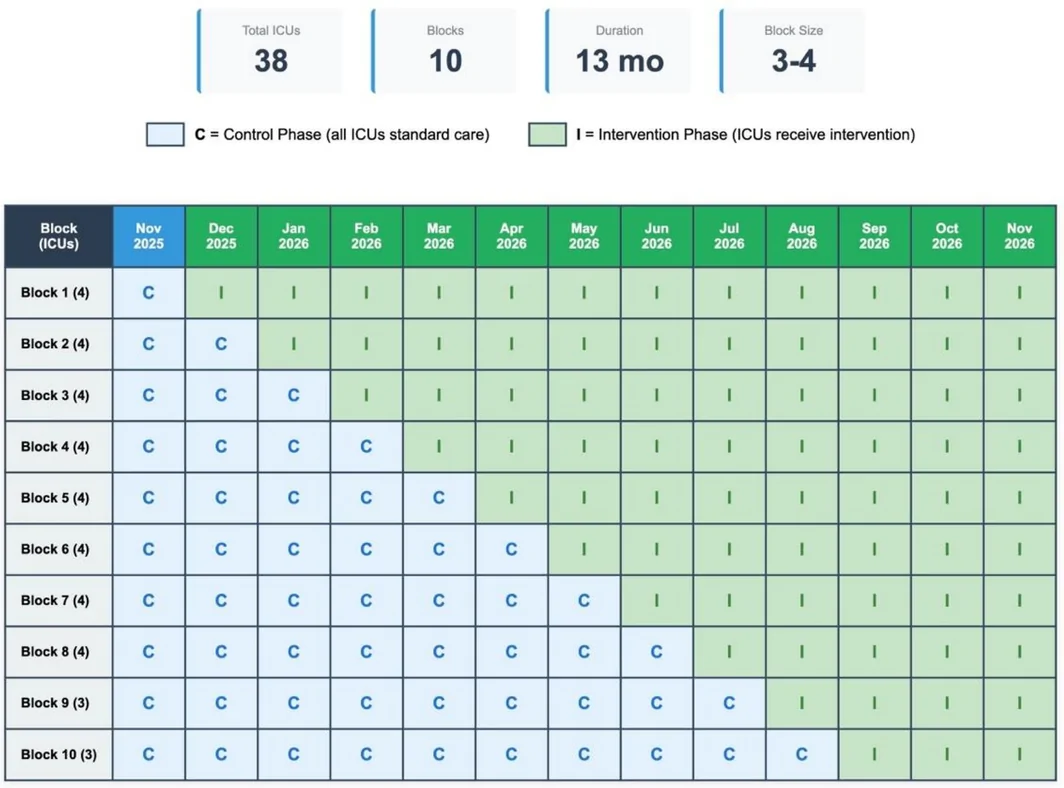
Randomization schedule.

WICH‐ICU is a registry‐based stepped‐wedge cluster randomized controlled trial [32]. We will roll out the intervention in 37 clusters (36 following the withdrawal of one unit after randomization; see Section 2.5). In all clusters, there will be a standard care phase and an implementation—intervention phase for a total of 13 months. The duration of each phase is determined by the randomized implementation sequence, but the standard care phase will be at least 1 month in all clusters. In this trial, clusters will be randomized to 1 of the 10 implementation sequences, meaning that three to four clusters will be randomized to each sequence (Figure 1). The schedule of enrollment, interventions, and assessments is shown in Table 1.

**TABLE 1:** Schedule of enrollment, interventions, and assessments (SPIRIT table).

	Baseline/Enrollment	Control period (Standard Care)	Intervention period (Choosing Wisely)
Timepoint	November 2025	December 2025–September 2026 (Months 1–10; varies by cluster)	December 2025–November 2026 (Months 1–13; varies by cluster)
Enrollment			
Cluster eligibility assessment	X		
Cluster randomization to sequence	X		
Patient eligibility (continuous)		X	X
Notification of cluster transition			X (6–8 weeks prior)
Interventions			
Standard care period	X	X	
*Choosing Wisely bundle*			
ABG protocol			X
Laboratory protocol			X
Chest x‐ray protocol			X
Assessments			
Diagnostic testing patterns (ABGs, laboratory tests and chest x‐rays)	X	X	X
Baseline characteristics (SAPS3)	X	X	X
Primary outcome	X	X	X
30‐day mortality	X	X	X
Secondary outcomes	X	X	X
ICU length of stay	X	X	X
Mechanical ventilation duration	X	X	X
Duration of CRRT	X	X	X
Number of unplanned (within 72 h) readmissions	X	X	X
Environmental impact	X	X	X

The stepped‐wedge design was selected for several reasons:

1. *Ethical considerations*: All participating sites eventually receive the intervention, addressing ethical concerns about withholding potentially beneficial interventions.
2. *Pragmatic implementation*: The design allows for staged implementation, facilitating training and resource allocation.
3. *Statistical efficiency*: Each cluster serves as its own control, increasing statistical power.
4. *Contamination minimization*: The temporal separation reduces the risk of contamination between the intervention and the control treatment.

### 3.2 Patient and Public Involvement

Patients and members of the public were not involved in setting the research question, designing the trial, or selecting the outcome measures. The intervention operates at the level of unit‐wide testing defaults rather than individual treatment decisions, no patient‐level recruitment or consent procedure exists, and all outcomes are ascertained from routinely collected registry data; the scope for meaningful individual‐level involvement in trial conduct was therefore limited. The choice of outcomes was nonetheless informed by patient‐relevant considerations: reduced iatrogenic anemia from cumulative diagnostic blood loss, reduced discomfort from repeated arterial punctures in patients without indwelling arterial lines, and reduced radiation exposure are among the documented benefits of the intervention, and the safety outcomes were selected as those a patient would care most about. Trial results will be disseminated to the public through SIR's and SFAI's public‐facing channels and through a plain‐language summary posted with the trial registration.

### 3.3 Setting and Participants

#### 3.3.1 Participating Sites

The study will be conducted in Swedish ICUs participating in the SIR. Eligible ICUs must:

- Have > 95% data completeness
- Treat adult patients (≥ 18 years)
- Have committed local leadership support
- Designate a clinical monitor for data collection

#### 3.3.2 Patient Population

All adult patients (≥ 18 years) admitted to participating ICUs during the study period will be included in outcome analyses. To account for the gradual onset of the treatment effect following the transition of each cluster from control to intervention, patients admitted within the first hour after the transition will be excluded from the analysis. No individual patient consent is required due to the registry‐based design and quality improvement nature of the interventions.

### 3.4 Recruitment

We approached all Swedish ICUs via SIR and SFAI (Svensk Förening för Anestesi och Intensivvård: the Swedish society for anesthesia and intensive care) using both their web pages and their email lists. ICUs were excluded if they had fully implemented the targeted CW recommendations since such units could not contribute a valid standard‐of‐care control period, or if they declined participation. The 38 units (37 clusters) randomized cover approximately 53% of all adult ICU admissions in Sweden; following the withdrawal of one small cardiothoracic unit after randomization, 37 units (36 clusters) contribute data, with coverage unchanged at approximately 53%. Individual patients are not recruited: all care episodes in participating units are identified through routine SIR registration, capturing over 95% of all Swedish ICU admissions using standardized definitions, and outcomes are ascertained by the registry. This registry‐based mechanism removes the risk of selective enrollment and is the reason no patient‐level recruitment procedures are required.

### 3.5 Intervention Bundle

The WICH‐ICU intervention consists of a bundle of three evidence‐based CW recommendations:

1. ABG Sampling Protocol

*Current practice*: Routine hourly ABG sampling in many ICUs.

*Intervention*: Structured protocol for ABG frequency:

- *Hourly sampling*: Unstable patients where results directly change treatment.
- *Every 2 h*: Unstable patients where results may not immediately change management.
- *Every 4 h*: Stable patients where results may change management.
- *Less frequently than every 4 h*: Stable patients where results are unlikely to change management.
  2 Blood test Protocol

*Current practice*: Daily routine laboratory panels regardless of clinical status.

*Intervention*: Risk‐stratified laboratory testing approach:

- *Daily testing*: Essential parameters (e.g., albumin, blood count, CRP, and creatinine)
- *Every other day/twice weekly*: Intermediate priority tests (e.g., liver enzymes, coagulation studies, electrolytes, and procalcitonin)
- *Avoid routine testing*: Low‐yield tests without specific clinical indication
  3 Chest Radiography Protocol

*Current practice*: Routine chest x‐rays after insertion of central venous lines, after insertion of nasogastric tubes, after initiation of invasive ventilation, and for many mechanically ventilated patients.

*Intervention*: Clinically indicated chest radiography only:

- Chest x‐rays performed *only when diagnostic information would directly affect clinical management*.
  ○ Examples of appropriate indications: suspected pneumothorax, line placement confirmation in “difficult” cases, acute respiratory deterioration.

### 3.6 Modification and Discontinuation of the Intervention

Because the intervention operates at the cluster level and modifies testing defaults rather than restricting indicated care, clinically indicated testing at any frequency constitutes intended use of the protocols rather than a deviation and requires no justification beyond routine clinical records. No concomitant care is prohibited, and no co‐intervention is introduced: all treatment other than the frequency of routine, non‐indicated diagnostic testing continues according to local practice in both trial conditions. The protocol materials and decision‐support tools distributed to participating units are available from the corresponding author on request.

At the cluster level, the intervention may be paused or modified at a participating ICU if the monthly safety monitoring identifies a signal of harm at that unit, if local circumstances (e.g., a major surge in patient volume or a staffing crisis) make continued adherence unsafe or impracticable, or at the request of local clinical leadership. Decisions to pause, modify, or discontinue the intervention at a site are made by the principal investigator together with the steering committee, in consultation with the local investigator and, where a safety signal is involved, the safety review group. Any such decision, its rationale, its duration, and the affected periods are documented in the trial master file, reported in the trial publication, and handled in the per‐protocol sensitivity analysis.

### 3.7 Implementation Strategy

#### 3.7.1 Pre‐Implementation Phase

- Baseline monitoring of current testing practices
- Staff education and training sessions
- Distribution of protocol materials and decision support tools
- Local champion identification and training

#### 3.7.2 Implementation Phase

- Phased rollout according to randomization schedule; all centers transitioning from control to intervention phase will be informed 6–8 weeks prior.
- Regular feedback on adherence and outcomes.
- Continuous quality improvement processes.

#### 3.7.3 Post‐Implementation Monitoring

- Ongoing assessment of protocol adherence, measured as the testing‐intensity outcomes defined under Secondary Estimands and analyzed as specified in the Statistical Analysis Plan.
- Safety monitoring and adverse event tracking.
- Periodic reinforcement and education sessions.
- Every month, data on overall mortality will be collected from the SIR and used to monitor deaths until the last patient has completed final follow‐up.

## 4 Outcomes

### 4.1 Primary Estimand

*Following the ICH E9(R1) estimand framework*:

The ICH E9(R1) addendum is the International Council for Harmonisation guideline on statistical principles for clinical trials, whose addendum (R1) defines the estimand framework used to provide a precise description of the treatment effect being estimated, including the handling of intercurrent events [33]. The primary estimand is defined as follows:

The population comprises adults (≥ 18 years) admitted to a participating ICU during the study period. The treatment conditions are management during an intervention period (the CW bundle) versus management during a control period (standard practice at each ICU). For patients with more than one ICU admission during the study period, each care episode is analyzed under the exposure condition prevailing at the time of that admission, and the resulting within‐patient correlation is accounted for in the analysis model.

The endpoint is all‐cause mortality within 30 days of ICU admission, ascertained through SIR's linkage to the Swedish population register. The population‐level summary measure is the adjusted absolute risk difference in 30‐day mortality (intervention minus control). Intercurrent events are expected to be rare: outcome ascertainment through the population register is essentially complete, and reversion of a cluster from intervention to standard care is not planned; intercurrent events are handled under a treatment‐policy strategy, consistent with the intention‐to‐treat principle.

### 4.2 Secondary Estimands

Secondary outcomes are defined per ICU care episode. ICU length of stay is the time from ICU admission to ICU discharge or death. The durations of invasive mechanical ventilation, noninvasive ventilation, and CRRT are the cumulative hours of each therapy during the ICU episode, as registered in SIR; because death precludes further accrual of these durations, they are analyzed in a time‐to‐event framework with death handled as a competing event. Unplanned ICU readmission is defined, per the SIR definition, as an unplanned return to intensive care within 72 h of ICU discharge, recorded as a binary outcome per discharged episode.

Testing‐intensity outcomes, referred to elsewhere in this protocol as process outcomes, are the number of ABG samples, venous laboratory tests, and chest radiographs per patient‐day.

The environmental outcome—climate impact expressed as carbon dioxide equivalents (CO_2_e, 100‐year global warming potential) attributable to blood gas sampling, laboratory testing, and chest radiography—will be estimated from the process‐outcome counts combined with published and supplier‐derived emission factors per test, with the functional unit being CO_2_e per patient‐day; a separate prespecified analysis plan will detail data sources, system boundaries, and methods, and this outcome is exploratory. Because death precludes the further accrual of tests and of their associated emissions, an intervention that increased early mortality would mechanically appear favorable on both testing intensity and CO_2_e. These outcomes are therefore subordinate to the primary safety outcome: they are interpreted as evidence of benefit only if non‐inferiority for 30‐day mortality is demonstrated, and if it is not, they are reported descriptively with no efficiency or environmental claim made. All such outcomes are expressed per patient‐day as well as per care episode, so that any shortening of the denominator through earlier death remains visible.

### 4.3 Outcome Measurement

*Registry data collection*: Primary and secondary outcomes will be collected through SIR, which captures standardized data elements for all Swedish ICU admissions, including:

- Demographics and comorbidities
- Severity of illness, Simplified Acute Physiology Score (SAPS 3)
- Interventions and procedures
- Outcomes and discharge status
- Unplanned readmission within 72 h

*Local monitoring*: Participating sites will collect process outcomes through designated clinical monitors during specified monitoring periods:

- Baseline period pre‐implementation (5 days)
- 1‐Month post‐implementation (5 days)
- 3‐Month post‐implementation (5 days)

#### 4.3.1 Validation of Local Monitoring

- High‐resolution electronic health data will be requested from clusters where available to allow more granular assessment of protocol adherence over the entire study period.
- An additional measure to validate the sampling performed by local clinical monitors is the documentation of departmental orders for vacutainer tubes and blood gas syringes.

Because a non‐inferiority design is vulnerable to a null result arising from non‐delivery rather than from true equivalence, intervention fidelity is treated as a precondition for interpreting the primary result. The fidelity criterion is an interpretive safeguard and not a definition of the analysis population: it does not determine which patients, clinicians, or clusters are analyzed, and the primary analysis remains an unconditional intention‐to‐treat comparison in which every care episode is analyzed according to the randomized state of its cluster at the time of admission, irrespective of local adherence. We prespecify that non‐inferiority will be interpreted as demonstrated only if the pooled reduction in testing intensity (ABG samples, venous laboratory tests, and chest radiographs per patient‐day) during intervention periods reaches at least 5% relative to the same clusters' control periods. The rationale is that a non‐inferiority finding on patient outcomes is informative only if the trial achieved a material separation between conditions: were testing to fall by, say, 1%, non‐inferior mortality would be near‐guaranteed irrespective of whether reduced testing is in fact safe. If the observed reduction falls below this threshold, the primary result will be reported as inconclusive with respect to the safety of CW implementation rather than as evidence of non‐inferiority. Because the criterion removes no care episode from the analysis population, it does not alter the power calculation.

### 4.4 Sample Size and Power Calculations

It is unlikely that the implementation of the CW protocol will lead to improved ICU outcomes [22, 24], so we wanted to avoid superiority metrics. Therefore, we opted for a non‐inferiority design. Pragmatic elements of the present study that we need to address:

- In Sweden, the CW concept is gaining traction, especially in the ICU setting. Thus, it is imperative that the present study is done rapidly. Some ICUs have already begun implementation of the CW concept and cannot be included.
- The present study would improve its power if it were performed over a 2‐year period, but this is not practically feasible, since both clinicians and policymakers advocate for a rapid implementation of CW.
- In order to more robustly assess the safety of CW, we will report multiple secondary outcomes in addition to the primary outcome: 30‐day mortality. ICU length of stay, time on invasive and noninvasive ventilation, time on CRRT, and unplanned readmissions add a high‐resolution layer of patient safety metrics.

With 38 ICUs included, covering 53% of all adult Swedish ICU admissions over the year (although this study runs for 13 months), the study findings will be broadly generalizable (37 ICUs following one withdrawal, as described under Section 2.5).

#### 4.4.1 Non‐Inferiority Power Analysis

We conducted a non‐inferiority‐based power calculation for our primary outcome of mortality, using a non‐inferiority margin of 3%. The analysis is based on data from the SIR.

The choice of a 3‐percentage‐point margin was made on the following grounds. First, the intervention removes routine, non‐indicated testing while explicitly preserving all clinically indicated diagnostics, and the aggregate randomized and observational evidence on diagnostic‐test reduction in intensive care shows no signal of increased mortality [22]; a true effect approaching the margin is therefore considered implausible a priori. Second, mortality is deliberately complemented by more sensitive safety outcomes that would be expected to deteriorate before any mortality effect became detectable, together with monthly safety monitoring throughout the trial. Third, the margin must be weighed against feasibility: demonstrating non‐inferiority within a substantially narrower margin (e.g., 1–2 percentage points) would require a trial several times larger, running over multiple years, which is not achievable while ICUs are already adopting CW practice outside trial conditions; the present design represents the strongest randomized evidence obtainable before uncontrolled implementation makes a controlled evaluation impossible. On balance, the steering committee judged that, for an intervention with documented benefits in reduced iatrogenic anemia, reduced resource use and reduced environmental impact, a 3‐percentage‐point margin represents the largest mortality difference that could be considered clinically acceptable, while the a priori expected difference is null. The margin may also be set against those adopted in recent critical care non‐inferiority trials with all‐cause mortality as the primary outcome. EVERDAC, comparing early with deferred arterial catheterization in shock, used a margin of 5 percentage points against a control‐group 28‐day mortality of 36.9% [34]. BALANCE, comparing 7 with 14 days of antibiotic therapy for bloodstream infection, used 4 percentage points against a control‐group 90‐day mortality of 16.1% [35]; three earlier trials in that field used 10 percentage points. In absolute terms, the present margin is smaller than both. In relative terms, 3 percentage points against a baseline 30‐day mortality of 18% corresponds to a risk ratio of 1.17, which lies between the corresponding figures for EVERDAC and BALANCE. The choice of margin does not by itself determine what would justify a change in practice; that question is addressed in the Discussion. Expressed in absolute terms, a true effect lying exactly at the margin would correspond to approximately 670 additional deaths across the projected 22,428 care episodes. The trial is therefore designed to exclude, rather than to detect, harm of this magnitude, and the secondary safety outcomes together with monthly monitoring are intended to identify smaller signals earlier.

#### 4.4.2 Power Calculation

Sample size was calculated using the swCRTdesign package in RStudio.

Parameters:

- *Stepped wedge design*: As shown in Figure 1.
- *Primary endpoint*: 30‐day mortality.
- *Design*: Non‐inferiority trial.
- *Number of patients per cluster and time period*: A total of 38 ICUs were included in the analysis. Two ICUs were merged into a single cluster because they share staff, resulting in *37 distinct clusters*. The stepped‐wedge design comprised *13 time periods*. Based on historical data, the total number of ICU admissions during the 13‐month study period was estimated to be *22,625 episodes*, derived from Swedish Intensive Care Registry admission data for the participating clusters over the corresponding calendar period [29]. Under these assumptions, the average number of ICU admissions per cluster and time period was calculated as 22,625 ÷ 13 ÷ 37 ≈ *47 admissions*.
- These are the parameters prespecified before recruitment opened. Following the withdrawal of one cluster, the corresponding figures are 22,428 episodes across 36 clusters and 13 periods, approximately 47 admissions per cluster‐period, a reduction of 0.9% that does not materially affect the power calculation, which has therefore not been repeated.
- *Alpha level*: 0.025 (one‐sided 97.5% CI).
- *Intraclass correlation coefficient (ICC)*: 0.009
  ○ The ICC was calculated using historic data via a generalized linear mixed model. To account for differences in baseline mortality across ICU types, the model included:
    - A variable distinguishing ICU category [1, 2, 3].
    - A variable indicating whether the ICU was a thoracic unit, which has distinct expected mortality rates.
- *Treatment effect was assumed to be homogeneous across clusters*.
- *No fixed time trends were assumed*.

*Non‐inferiority margin assumption for 30‐day mortality*: Based on historical data, the mean baseline 30‐day mortality across the relevant clusters was estimated at 18%, based on Swedish Intensive Care Registry historical data for the participating clusters [29]. The non‐inferiority margin was defined as an absolute risk difference of 3 percentage points. Assuming the same baseline mortality, this corresponds to a relative risk of 1.17 and an odds ratio of approximately 1.21.

*Power to demonstrate non‐inferiority under the assumptions stated above*: 89%. A Power > 80% will be considered acceptable to demonstrate non‐inferiority.

### 4.5 Randomization and Allocation

#### 4.5.1 Cluster Definition

Individual ICUs represent clusters for randomization purposes, with one exception: where two ICUs have physicians and nurses working at both ICU sites in the same hospital. These two interconnected ICUs are seen as one cluster. SIR categorizes ICUs into Levels 1, 2, and 3 based on care level [36].

ICUs in Category 1 manage intensive care for patients with failure in all organ systems but lack most supporting resources. The hospital lacks access to most specialties and resources around the clock, for example, pediatrics, vascular surgery, ENT surgery, and blood bank. ICUs in Category 1 should be able to consult and send patients for care at ICUs in Categories 2 and 3. For this to work, ICUs in Category 1 must be able to receive patients with less complex needs from Category 2 and 3 units.

ICUs in Category 2 manage intensive care for patients with failure in all organ systems but lack Category 3's supporting resources. The hospital can still function as a referral center for the region, for example, for specialties such as pediatrics, vascular surgery, angiographic services, and/or ENT surgery. They therefore have access to most specialties, particularly emergency surgery, radiology, laboratory, and blood bank around the clock. ICUs in Category 2 should be able to consult and send patients for care at ICUs in Category 3. For this to work, units within Category 2 must be able to receive patients with less complex needs from Category 3 units.

Category 3 consists primarily of ICUs at university hospitals. These provide the most advanced monitoring and treatment methods for various types of organ failure. Since this category typically represents the full capacity of Swedish healthcare, they serve as referral centers for smaller hospitals. ICUs within Category 3 should have a system to oversee all the hospital's intensive care resources and have clear communication pathways into the hospital, preferably with a lead intensive care physician who can be reached on a dedicated phone line from referring hospitals. To function as a referral center with preparedness to receive the most complex patients regardless of place of residence, ICUs in Category 3 must also be able to transfer less complex intensive care patients to Category 1 and 2 units in the surrounding area.

#### 4.5.2 Randomization Scheme

The 37 clusters were randomized to 1 of the 10 implementation sequences, with three or four clusters allocated per sequence; this restricted allocation ensures a balanced stepped‐wedge layout across the 13 monthly periods. The allocation sequence was generated by a constrained randomization algorithm implemented in R (version 4.6) with a fixed random seed. The seed, the generating script, and equivalent implementations in Stata and Python have been archived and will be made available with the trial report, allowing full reproduction of the allocation. Randomization was stratified by ICU category and ICU size (admissions/year). One prespecified constraint was applied: the two ICUs sharing staff at the same hospital were treated as a single cluster and therefore necessarily follow the same sequence. The sequence was created by Max Bell, who had no clinical role at any participating unit. Because randomization was at the cluster level and no individual patient was enrolled, there were no enrolling personnel whose behavior could be influenced by foreknowledge of the next allocation, and conventional allocation concealment is not applicable in the individual‐participant sense. The generated sequence was not disclosed to participating units before allocation was complete; each cluster was subsequently informed of its own transition date 6–8 weeks in advance, as described above. The full allocation list is stored securely with the trial master file.

## 5 Statistical Analysis Plan

### 5.1 General Principles

- Analysis of the primary outcome follows the intention‐to‐treat principle, with every care episode analyzed according to the randomized state of its cluster at the time of admission and irrespective of local adherence; a prespecified per‐protocol analysis is reported alongside it.
- Mixed‐effects models will account for clustering and temporal trends.
- Multiple imputation will address missing data when > 10% are missing for key covariates.
- Sensitivity analyses will explore the robustness of findings.
- Statistical significance will be set at *α* = 0.05 for superiority analyses and *α* = 0.025 (one‐sided) for non‐inferiority analyses.

No adjustment for multiplicity is made. Only the primary outcome supports a confirmatory conclusion; secondary, process, and exploratory outcomes are reported as point estimates with 95% confidence intervals (CIs) and interpreted as supportive, with no formal non‐inferiority testing applied to them.

### 5.2 Primary Analysis—Mortality (Non‐Inferiority)

The primary analysis fits a generalized linear mixed‐effects model for 30‐day mortality with a binomial distribution and logit link. The model includes a fixed effect for treatment condition (intervention versus control), fixed effects for calendar time modeled as categorical monthly periods, fixed‐effect adjustment for the prespecified baseline covariates (SAPS 3 score, age, sex, admission type [emergency vs. planned], and ICU category [SIR Levels 1–3]), and a random intercept for cluster, implying an exchangeable within‐cluster correlation structure. Sensitivity analyses will explore alternative correlation structures, including a random cluster‐by‐period effect allowing the within‐period correlation to exceed the between‐period correlation. Because a minority of patients contribute more than one care episode, the model also includes a random intercept for patient nested within cluster. A sensitivity analysis restricted to each patient's first ICU admission during the study period will be reported.

Because the estimand is an absolute risk difference, adjusted marginal risks under intervention and control are obtained from the fitted model by regression standardization (g‐computation): the model is used to predict each episode's outcome probability under both treatment conditions, and these predictions are averaged over the trial population. The adjusted risk difference is the difference between the two standardized risks, with a 95% CI obtained by cluster bootstrap or delta method. Non‐inferiority is declared if the upper bound of the two‐sided 95% CI (equivalently the one‐sided 97.5% bound) for the adjusted risk difference (intervention minus control) lies below 3 percentage points. Intention‐to‐treat analysis behaves differently in a non‐inferiority trial than in a superiority trial. Whereas incomplete adherence biases a superiority comparison toward the null and is therefore conservative, in a non‐inferiority comparison it makes the two conditions more alike and so makes a conclusion of non‐inferiority easier to reach; intention‐to‐treat is in this sense anti‐conservative [37]. We therefore prespecify a per‐protocol analysis alongside the primary intention‐to‐treat analysis. The per‐protocol population comprises all control periods together with those intervention cluster‐periods in which the cluster's own testing intensity met the fidelity criterion; cluster‐periods during which the intervention was paused or modified under the provisions above are excluded from the intervention condition. The per‐protocol analysis uses the same model, covariates, and non‐inferiority margin as the primary analysis. A conclusion of non‐inferiority requires the upper confidence bound to lie below the margin in both analyses. Where the two are discordant, both are reported, and the discordance is discussed rather than resolved by rule. Realized testing intensity is reported for both conditions at cluster level so that the separation achieved is visible to the reader.

ICU length of stay and the durations of invasive ventilation, noninvasive ventilation, and CRRT are analyzed in a time‐to‐event framework, with time origin at ICU admission (for length of stay) or at initiation of the respective therapy (for therapy durations). The events of interest are, respectively, live ICU discharge and liberation from the therapy; death before the event is treated as a competing event using cause‐specific Cox proportional‐hazards models with a gamma‐distributed cluster‐level frailty to account for clustering, complemented by cause‐specific cumulative incidence functions. Episodes are administratively censored at 30 days from ICU admission for length of stay, and from initiation of the respective therapy for the durations of invasive ventilation, noninvasive ventilation, and CRRT; episodes still ongoing at that point are censored. Treating death as a competing event rather than censoring avoids the bias whereby early deaths masquerade as short, favorable stays. Proportional‐hazards assumptions are assessed with Schoenfeld residuals; where violated, effects are reported as time‐varying or summarized as restricted‐mean‐survival‐time differences. Models are adjusted for the same covariates as the primary analysis. Unplanned ICU readmission within 72 h is analyzed among live ICU discharges with a logistic generalized linear mixed‐effects model specified analogously to the primary analysis.

### 5.3 Process Measures

Testing intensity is analyzed, not solely as a fidelity check but as a prespecified outcome. For each targeted category—ABG samples, venous laboratory tests, and chest radiographs—counts per patient‐day are analyzed in a mixed‐effects negative binomial model with a log link, an offset for observed patient‐days, a random intercept for cluster, and fixed effects for calendar period and treatment condition, specified analogously to the primary analysis. Effects are reported as incidence rate ratios with 95% CIs, separately by category and pooled across the three. Because these counts are ascertained from 5‐day monitoring windows at baseline and at 1 and 3 months after transition rather than continuously, the estimates describe testing intensity during sampled windows and are reported as such. Importantly, where high‐resolution electronic health record data are available, a supporting analysis using continuous data across the full study period is reported for those clusters and its agreement with the sampled estimates described. Departmental orders for vacutainer tubes and blood‐gas syringes are reported at cluster‐period level as a third, independent indicator of testing volume.

### 5.4 Handling of Clustering and Temporal Effects

#### 5.4.1 Stepped‐Wedge Design Considerations

- Random effects for clusters to account for unmeasured cluster‐level confounders.
- Sensitivity analyses using different correlation structures.

#### 5.4.2 Handling of Missing Data

- Complete case analysis as primary approach given high data completeness in the SIR (> 95%).
- Multiple imputation using chained equations if missing data > 10% for key variables.
- Missing data patterns will be assessed and reported.
- Sensitivity analyses comparing complete case and imputed results.

### 5.5 Sensitivity Analyses

1. *Time trend specifications*: Alternative modeling of temporal trends.
2. *Outlier ICUs*: To assess the influence of potentially outlying clusters, a binary indicator variable identifying these clusters will be included in the model both as a main effect and in interaction with treatment.
3. *Baseline period*: Varying length of baseline observation period.
4. *Minimal detectable difference*: Minimal detectable difference will be calculated to evaluate the assumptions of power analysis.
5. To assess for the gradual onset of the treatment effect following each cluster's transition from control to intervention, the treatment variable will be replaced by a categorical variable with the following levels: “Before switch,” “0–7 days after switch,” “8–14 days after switch,” and “> 14 days after switch.”
6. If ICU drop‐out or wrongful randomizations occur, a per‐protocol analysis will be performed to account for this.
7. Contemporaneous external comparison: Because SIR captures over 95% of Swedish ICU admissions, admissions to nonparticipating Swedish ICUs over the same calendar months provide a contemporaneous, non‐randomized external comparison. Risk‐adjusted 30‐day mortality is compared between participating and nonparticipating units by calendar period, adjusted for SAPS 3, age, sex, admission type, and ICU category, to characterize secular trend and background fluctuation in mortality unrelated to the intervention and to provide an indirect check for diffusion of CW practice beyond the trial. Because participation was not randomly assigned and participating units differ from nonparticipating units in size, category, and case mix, this comparison is descriptive and is not interpreted as an effect estimate.

### 5.6 Subgroup Analyses

Prespecified subgroup analyses will examine intervention effects across:

- ICU Category 1, 2, and 3 according to the SIR definitions.

### 5.7 Blinding

Because the intervention changes clinical routines at the unit level, ICU personnel, treating physicians, and local investigators cannot be blinded to allocation status, and no attempt at blinding of clinical staff is made. Patients are not actively informed of their unit's phase but are not formally blinded. No blinding of registry personnel is claimed; however, the primary outcome—30‐day all‐cause mortality—is ascertained automatically through SIR's linkage to the Swedish population register, and the secondary outcomes are extracted from routinely registered SIR data whose collection is identical in control and intervention periods and independent of the trial. Outcome ascertainment is therefore objective and not susceptible to assessor bias, which is the principal protection against detection bias in this open‐label design.

Data management and statistical analysis will be performed with the treatment condition held as a coded exposure variable until the analysis code is finalized, so that the analyst is blinded to allocation during code development; the statistical analysis code will be finalized and archived before the exposure coding is revealed. Because the trial is open‐label at the cluster level, there is no individual participant allocation that could be revealed, and no unblinding procedure is required. Follow‐up for the primary outcome is complete by construction: 30‐day mortality is ascertained through SIR's linkage to the Swedish population register rather than through participant contact, so loss to follow‐up is not a meaningful mechanism in this trial.

### 5.8 Safety Monitoring

A formal data monitoring committee with interim efficacy analyses is not established, which we consider justified by the pragmatic registry‐based design, the minimal‐risk nature of an intervention that removes only non‐indicated routine testing while preserving all clinically indicated diagnostics, and the absence of any planned early stopping for benefit. Safety oversight is instead provided by an independent safety review group comprising Ritva Kiiski Berggren and Johnny Hillgren, both senior intensivists with deep knowledge of SIR data, none of whom are trial investigators.

The group reviews unblinded monthly reports generated from SIR, comprising 30‐day mortality and the secondary safety outcomes by cluster and by intervention status, until the last patient has completed follow‐up. The group may recommend continuation, modification, suspension of the intervention at individual ICUs, or termination of the trial; recommendations are made to the principal investigator and steering committee, who are responsible for acting on them and for informing participating ICUs and, where required, the Swedish Ethical Review Authority. If interim monitoring indicates increasing mortality or adverse changes in any secondary safety outcome, the affected ICU will be contacted to discuss appropriate action. A quantitative trigger is prespecified. Review by the safety review group is initiated whenever a cluster's observed 30‐day mortality during its intervention periods exceeds the upper bound of the 95% prediction interval derived from that cluster's own historical SIR mortality for the corresponding calendar months, or whenever the pooled intervention‐versus‐control mortality difference on the cumulative monthly report exceeds 3 percentage points. Meeting a trigger prompts review and, at the group's discretion, contact with the affected unit and a recommendation to the steering committee; it does not automatically suspend the trial.

### 5.9 Harms

The conceivable harms of reducing routine diagnostics are delayed recognition of clinical deterioration, of laboratory derangements such as electrolyte disturbance or occult bleeding, or of mechanical complications such as pneumothorax or catheter malposition that routine imaging might otherwise detect. Because the trial is embedded in routine care and modifies testing defaults rather than introducing a therapeutic agent, harms are monitored at two levels. At the population level, the monthly safety reports track 30‐day mortality and the secondary safety outcomes by cluster and intervention status; these outcomes are deliberately chosen as sensitive upstream indicators that would deteriorate before mortality.

At the patient level, clinicians and local investigators are instructed to report any serious adverse event suspected to be related to reduced testing, in particular missed or delayed diagnoses with clinical consequences, to the principal investigator without undue delay after becoming aware of it. Causality is assessed by the independent safety review group, which reviews the case documentation and classifies events as unrelated, possibly related, or probably related to the intervention. Confirmed or suspected intervention‐related serious harms and any safety signal in the monthly reports trigger review by the safety review group, which may recommend pausing the intervention at the affected ICU or across the trial; such events are reported to the steering committee, the affected ICU, and, where required under Swedish regulations, the Swedish Ethical Review Authority. All reported events and actions taken are summarized in the trial publication. Patients are covered by the ordinary Swedish patient‐injury insurance (patientförsäkringen, LÖF).

### 5.10 Reporting Standards

Results will be reported according to:

- SPIRIT statement [38] for the reporting of this protocol, with the completed SPIRIT checklist supplied as a Supplementary File S1.
- CONSORT extension for cluster randomized trials.
- CONSORT extension for stepped‐wedge cluster randomized trials.
- Complete statistical analysis code will be made available.

### 5.11 Data Management and Quality Assurance

#### 5.11.1 Data Collection and Management

##### 5.11.1.1 Data Sources

*Primary Data Source—SIR*: All patient outcome data will be extracted from SIR, which uses secure servers. The data includes:

##### 5.11.1.2 Demographic Data

- Unit name, number of ICU beds, and ICU category
- Sex and age
- Comorbidities
- Admission time and discharge time
- Care episode ID (for SIR internal use only)
- Route of admission and reason for admission
- Emergency admission status and surgical status
- Mortality and time of death
- Parent clinic

##### 5.11.1.3 Severity of Illness Assessment

- *SAPS3* [39] including all component variables, total score, and estimated mortality risk

##### 5.11.1.4 Treatment and Interventions

- All treatment strategy variables
- All care intensity measurement variables
- Mechanical ventilation (invasive and noninvasive) duration
- Renal replacement therapy duration and type
- Complication codes
- Care limitations and withdrawal decisions

##### 5.11.1.5 Outcomes

- 30‐day mortality
- ICU length of stay
- Unplanned ICU readmission within 72 h from discharge
- Mortality data with precise timing.

*Process measures—Local monitoring*: Clinical monitors will study the utilization/frequency of ABGes, venous laboratory testing, and chest radiography during both control and intervention phases. Since this data is not registered in SIR, an electronic Case Report Form (eCRF) will be created to capture:

- Number of ABG samples per patient‐day
- Number of venous laboratory tests per patient‐day
- Number of chest x‐rays per patient‐day
- Protocol adherence measures

#### 5.11.2 Data Quality and Validation

Data quality and protocol adherence are audited on two levels. SIR performs its ordinary registry‐wide validation of reported data, independent of the trial. In addition, trial‐specific audits are conducted by the investigators in conjunction with the local monitors, in which locally collected process data (eCRF entries for blood‐gas, laboratory and radiography counts) are cross‐checked against source data in two forms: the electronic health record and departmental consumables orders. Audit findings are documented in a standardized report to the steering committee; discrepancies trigger retraining of the local monitor and, where systematic, correction of the affected data, with corrective actions followed up at the subsequent audit. Local monitors receive standardized training on data‐collection protocols before site initiation, and data completeness is monitored continuously.

#### 5.11.3 Data Security

- SIR manages secure servers.
- All data will be handled according to Swedish data protection regulations.
- Personal identifiers will be removed for analysis datasets.
- Secure data transfer protocols will be implemented.

## 6 Ethical Considerations

### 6.1 IRB/Regulatory Approval

The study has received approval from the Swedish Ethical Review Authority (reference numbers: 2025‐01558‐01, 2025‐06782‐02, and 2025‐07290‐02) and will be conducted in accordance with the Declaration of Helsinki.

### 6.2 Protocol Amendments

Protocol amendments are proposed to and approved by the steering committee. Substantive amendments—those affecting eligibility, the intervention, the outcomes, the analysis plan, or patient safety—are submitted to the Swedish Ethical Review Authority for approval before implementation, recorded in an updated protocol version with the date, the change, and its reason, reflected in the ClinicalTrials.gov record, and communicated to all participating units and to the independent safety review group. Non‐substantive administrative changes are recorded in the trial master file. All amendments will be reported in the trial publication.

### 6.3 Consent to Participate

The Swedish Ethical Review Authority granted a waiver of the requirement for individual informed consent under the approvals cited above. The grounds were:

- Registry‐based design using existing data collection systems
- Quality improvement nature of interventions
- Minimal risk profile of interventions
- Impracticality of individual consent in ICU settings

Patients retain the ordinary right under Swedish law to decline registration in national quality registries, and that right is unaffected by the trial. No biological specimens are collected for trial purposes, and the trial uses only data already recorded during routine care and registered in the SIR. No ancillary studies requiring separate consent provisions are planned; any future ancillary use of trial data would require amendments or a separate application to the Swedish Ethical Review Authority.

### 6.4 Risk–Benefit Assessment

No trial‐specific post‐trial intervention or ancillary‐care arrangement is planned, because the trial evaluates a cluster‐level modification of routine ICU practice rather than an individual‐level therapy. After the trial, patients continue to receive ordinary intensive care according to local routines, and each participating ICU decides locally, informed by the trial results and its own leadership, whether to continue, adapt, or discontinue the CW protocols; no automatic adoption is implied. Sweden's ordinary patient‐injury and compensation arrangements (patientförsäkringen) apply throughout and after the trial, and no trial‐specific compensation mechanism exists.

## 7 Discussion

The WICH‐ICU trial will address several important knowledge gaps in critical care and implementation science.

The WICH‐ICU trial will provide randomized evidence on the safety of systematically implementing CW recommendations in intensive care, an area where high‐quality evidence is currently scarce [22, 24]. By pairing 30‐day mortality with a set of more sensitive safety outcomes, and by embedding the intervention in routine care across a national registry, the trial is designed to detect any safety signal while remaining pragmatic and generalizable.

The design has several strengths. The stepped‐wedge approach maximizes statistical efficiency while ensuring that every site eventually receives the intervention; the registry‐based approach leverages a high‐quality, near‐complete national data infrastructure; cluster randomization minimizes contamination and reflects real‐world implementation; and the multi‐site design, covering roughly half of Swedish adult ICU admissions, enhances generalizability. The interventions are grounded in established professional recommendations, are designed for routine clinical use, and are supported by a local‐champion model that promotes sustainability.

The non‐inferiority margin defines the decision rule for a prespecified statistical hypothesis; it is not the threshold at which we would recommend a change in practice. Because the intervention removes routine testing rather than adding treatment, and because the benefits at stake are efficiency and environmental rather than clinical, the trade‐off is asymmetric: a mortality difference lying well within the margin could still be judged unacceptable for an intervention of this kind. We will therefore report the point estimate and the full confidence interval for the adjusted risk difference alongside the formal non‐inferiority conclusion, and we state in advance that a point estimate indicating excess mortality under the intervention would not, in our view, support de‐implementation of the targeted diagnostics even were the upper confidence bound to fall below 3 percentage points. As noted above, a true effect at the margin would correspond to approximately 670 additional deaths across the projected care episodes; a difference half that size would remain clinically material and will be reported as such.

Several limitations warrant consideration. Secular trends may confound intervention effects, although calendar time is modeled explicitly; registry data may not capture all relevant confounders; local implementation may vary between sites; and knowledge of the intervention could influence practice during control periods. As a single‐country study, the results may not fully generalize to healthcare systems with different practice patterns, culture, or resources.

Beyond patient safety and resource use, the trial will quantify the environmental impact of reducing low‐value testing, contributing to an emerging evidence base that links CW to healthcare sustainability [26, 40]. The trial does not undertake a formal health‐economic evaluation, and we make no claim about monetary savings. Most of the short‐run cost of a blood gas, a laboratory panel or a chest radiograph is staff time and installed capacity, both fixed over the horizon of this trial; reduced test volume does not become reduced expenditure unless that capacity is redeployed, and internal cross‐charging between clinical and service departments can offset an apparent saving within the same system. Reductions in testing are therefore reported in natural units and should not be read as realized savings. The environmental outcome is less exposed to this argument, since avoided consumables, reagents, and their transport are variable inputs whose emissions are avoided directly, and it is for this reason that CO_2_e rather than cost is reported as the efficiency‐side outcome. If non‐inferiority is demonstrated, the results will support evidence‐based de‐implementation of low‐value ICU diagnostics, and the design may serve as a model for similar evaluations in other settings and clinical areas.

## Author Contributions

Conceptualization of the project: Max Bell. Study design: Max Bell, Emma Larsson, Erik Svensk, Jesper Eriksson, and Arman Valadkhani. Statistical analysis plan: Max Bell, Emma Larsson, Jesper Eriksson, and Arman Valadkhani. Project lead: Max Bell. Steering committee: Fredrik Hammarskjöld, Markus Castegren, Peter Bentzer, Nicklas Nielsen, Mikael Eriksson, and Kristina Svennerholm. Read and approved the final manuscript: all authors.

## Funding

The authors have nothing to report.

## Disclosure

The trial received no external funding, so no funder had any role in its design or conduct. The sponsor had no role in trial design, data collection, analysis, interpretation, manuscript preparation or the decision to submit for publication, and holds no right to review or comment on the manuscript before submission. Final decisions on all trial matters rest with the principal investigator and the steering committee.

## Ethics Statement

The authors have nothing to report.

## Consent

The authors have nothing to report.

## Conflicts of Interest

The authors declare no conflicts of interest.

## Supporting information


**Data S1:** SPIRIT 2025 checklist—WICH‐ICU.

## Data Availability

The data analyzed in this study derive from Swedish national registers. Individual‐level data can only be accessed through secure servers, and only aggregated data, as presented in research articles, may be exported. Access requires fulfillment of specific requirements safeguarding participant anonymity and data security. For these reasons, the data are not publicly available. The statistical analysis code, the randomization script, and the random seed will be made available with the trial report.
