# Supplementary material for: WIse CHoices in the ICU (WICH‐ICU): Protocol and Statistical Analysis Plan for a Stepped‐Wedge Cluster Randomized Trial of Choosing Wisely Interventions in Swedish Intensive Care Units
Source: Acta Anaesthesiol Scand. 2026 Sep 23;70(10):e70339. doi: 10.1111/aas.70339 (PMC13600950; doi:10.1111/aas.70339)
Supplement: Supplementary file 1 [file AAS-70-0-s001.docx]

**SPIRIT 2025 checklist — WICH-ICU**

*WIse CHoices in the ICU (WICH-ICU): Protocol and Statistical Analysis Plan for a Stepped-Wedge Cluster Randomized Trial of Choosing Wisely Interventions in Swedish Intensive Care Units*

ClinicalTrials.gov NCT07013175 · Corresponding author: Max Bell · Page numbers refer to the submitted manuscript.

*All 34 items are addressed in the manuscript at the pages shown, or are explicitly stated as not applicable with a justification. Page numbers refer to the 26-page submitted manuscript.*

| **Section / Topic** | **No** | **SPIRIT 2025 checklist item description** | **Where addressed in the WICH-ICU protocol** |
| --- | --- | --- | --- |
| **Administrative information** | | | |
| Title and structured summary | **1a** | Title stating the trial design, population, and interventions, with identification as a protocol | Page 1. Title states the stepped-wedge cluster randomized design, the Swedish ICU population, the Choosing Wisely intervention, and identifies the document as a protocol and statistical analysis plan. The non-inferiority framework is stated in the Abstract (page 2) and in Sample Size and Power (page 11). |
|  | **1b** | Structured summary of trial design and methods, including items from the WHO Trial Registration Data Set | Pages 2 (structured Abstract: design, population, intervention, primary and secondary outcomes) and 5 (Trial registration and Trial status: registry, identifying number and URL, recruitment dates). Remaining WHO Trial Registration Data Set items appear elsewhere in the manuscript: primary sponsor and contacts page 24, target sample size page 12, ethics review page 22, and the participant data sharing statement page 24. |
| Protocol version | **2** | Version date and identifier | Page 5 (Trial status). Gives the current protocol version and date and the date of version 1.0, together with a summary of the changes made in version 2.0 — the pre-specified fidelity threshold, the quantitative safety-review trigger, and expanded reporting of trial oversight and open-science provisions — and states that no changes were made to eligibility, the intervention, the outcomes or the primary analysis. |
| Roles and responsibilities | **3a** | Names, affiliations, and roles of protocol contributors, including use of artificial intelligence technologies, if applicable | Pages 1 (author list and affiliations), 24 (Author contributions), 24 (Use of AI-assisted technology). |
|  | **3b** | Name and contact information for the trial sponsor | Page 24 (Sponsor and trial oversight). The trial sponsor is Karolinska Institutet, Stockholm, Sweden, with enquiries directed to the principal investigator. |
|  | **3c** | Role of trial sponsor and funders in design, conduct, analysis, and reporting; including any authority over these activities | Page 24 (Funding) and page 24 (Sponsor and trial oversight: Role of the sponsor and funders). |
|  | **3d** | Composition, roles, and responsibilities of the coordinating site, steering committee, endpoint adjudication committee, data management team, and other groups overseeing the trial | Pages 19 (independent safety review group: named members, role, reporting structure and independence), 24 (Sponsor and trial oversight: steering committee composition, executive authority and coordinating site) and 24 (Author contributions). |
| **Open science** | | | |
| Trial registration | **4** | Name of trial registry, identifying number (with URL), and date of registration | Pages 2 and 5: ClinicalTrials.gov, NCT07013175, https://clinicaltrials.gov/study/NCT07013175, first posted 2025-06-17. |
| Protocol and statistical analysis plan | **5** | Where the trial protocol and statistical analysis plan can be accessed | Page 5 (Trial status). States that this publication constitutes both the trial protocol and the statistical analysis plan, that the full versions of both are deposited with the trial registration record, and that they are available from the corresponding author on request. |
| Data sharing | **6** | Where and how the individual de-identified participant data, statistical code, and any other materials will be accessible | Pages 20 (complete statistical analysis code will be made available) and 24 (Data sharing statement, setting out why individual-level Swedish registry data cannot be exported and the conditions under which access may be granted). |
| Funding and conflicts of interest | **7a** | Sources of funding and other support (e.g., supply of drugs) | Page 24 (Funding). The trial received no external funding and uses existing registry infrastructure and routine clinical resources. |
|  | **7b** | Financial and other conflicts of interest for principal investigators and steering committee members | Pages 25 (Disclosures) and 24 (Sponsor and trial oversight), which states that neither the steering committee nor the safety review group members have financial or other conflicts of interest to declare. |
| Dissemination policy | **8** | Plans to communicate trial results to participants, healthcare professionals, the public, and other relevant groups; authorship guidelines | Page 5 (Trial status), which sets out publication regardless of the direction of findings, posting of results to the trial registry, communication to participating ICUs and the wider Swedish intensive care community through SIR and SFAI, a plain-language summary posted with the trial registration, and ICMJE authorship criteria with no professional medical writers engaged. See also page 20 (reporting according to the CONSORT cluster and stepped-wedge extensions). |
| **Introduction** | | | |
| Background and rationale | **9a** | Scientific background and rationale, including summary of relevant studies examining benefits and harms for each intervention | Pages 3–5. Includes the rationale for Choosing Wisely in intensive care, the evidence for each of the three bundle components, the identified knowledge gaps, and the Swedish healthcare context. |
|  | **9b** | Explanation for choice of comparator | Pages 6–7 and 15. The comparator is each cluster's own standard care during its control period, with each ICU serving as its own control. Usual care is measured rather than assumed, through the process outcomes collected in both trial conditions (page 10). |
| Objectives | **10** | Specific objectives related to benefits and harms | Pages 5 (Primary and Secondary Objectives, Hypotheses), 9 (Primary Estimand, following ICH E9(R1)), 10 (Secondary Estimands) and 19 (Harms). |
| **Methods: Patient and public involvement, trial design** | | | |
| Patient and public involvement | **11** | Details of, or plans for, patient or public involvement in the design, conduct, and reporting of the trial | Page 7 (Patient and public involvement). States that patients and members of the public were not involved in setting the research question, designing the trial or selecting the outcomes, with the reasons; notes that the choice of outcomes was nonetheless informed by patient-relevant considerations including iatrogenic anaemia, discomfort from repeated arterial puncture and radiation exposure; and sets out how results will be disseminated to the public. |
| Trial design | **12** | Description of trial design including type of trial, allocation ratio, and framework | Pages 5–6 (registry-based stepped-wedge cluster randomised design, 37 clusters, 13 monthly periods, 10 implementation sequences, 3–4 clusters per sequence), 11 (non-inferiority framework) and 15 (ICU as the unit of randomisation). |
| **Methods: Participants, interventions, and outcomes** | | | |
| Trial setting | **13** | Settings and locations where the trial will be conducted | Page 6. 38 Swedish intensive care units (37 clusters) participating in the Swedish Intensive Care Registry, covering approximately 53% of all adult Swedish ICU admissions. |
| Eligibility criteria | **14a** | Eligibility criteria for participants | Page 7 (Patient Population). All adults aged ≥18 years admitted to a participating ICU during the study period; episodes beginning within the first hour after a cluster's transition are excluded. |
|  | **14b** | If applicable, eligibility criteria for sites and for individuals who will deliver the interventions | Pages 6–7 (ICU eligibility: >95% data completeness, adult patients, committed local leadership, a designated clinical monitor) and 7 (exclusion of units that had already fully implemented the recommendations, and of units declining participation). |
| Intervention and comparator | **15a** | Intervention and comparator with sufficient detail to allow replication, including how, when, and by whom they will be administered; where additional materials can be accessed | Pages 8–9. All three bundle components are specified: the arterial blood gas sampling frequency protocol, the risk-stratified laboratory testing protocol, and the clinically-indicated-only chest radiography protocol, each contrasted against current practice. |
|  | **15b** | Criteria for discontinuing or modifying the allocated intervention/comparator for a participant | Pages 8–9 (Modification and discontinuation of the intervention). Clinically indicated testing at any frequency constitutes intended use rather than deviation; cluster-level pause, modification and discontinuation criteria and the decision process are specified. |
|  | **15c** | Strategies to improve adherence to intervention/comparator protocols and procedures for monitoring adherence | Pages 9 (implementation strategy: education, decision-support tools, local champions, feedback, 6–8 weeks' advance notification) and 10–11 (local monitoring periods, validation against EHR extracts and departmental consumables orders, and the pre-specified fidelity threshold below which the primary result is reported as inconclusive rather than as evidence of non-inferiority). |
|  | **15d** | Concomitant care that is permitted or prohibited during the trial | Page 9 (Modification and discontinuation of the intervention): no concomitant care is prohibited and no co-intervention is introduced; all treatment other than the frequency of routine, non-indicated diagnostic testing continues according to local practice in both trial conditions. |
| Outcomes | **16** | Primary and secondary outcomes, including the measurement variable, analysis metric, method of aggregation, and time point for each | Pages 9–10. The primary estimand specifies 30-day all-cause mortality with the adjusted absolute risk difference as the population-level summary. Secondary estimands specify ICU length of stay, invasive and non-invasive ventilation duration, CRRT duration and unplanned readmission within 72 hours, each with its metric and time frame. Process outcomes and the exploratory environmental outcome are defined on page 10. |
| Harms | **17** | How harms are defined and will be assessed | Page 19 (Harms). Specifies the conceivable harms of reduced diagnostics, population-level monitoring through the monthly safety reports, patient-level reporting of serious adverse events suspected to relate to reduced testing, causality assessment by the independent safety review group, and the reporting pathway. |
| Participant timeline | **18** | Time schedule of enrolment, interventions, assessments, and visits; a schematic diagram is highly recommended | Page 14 (Table 1, Schedule of Enrolment, Interventions, and Assessments) and page 6 (Figure 1, randomisation schedule showing the stepped-wedge rollout). |
| Sample size | **19** | How sample size was determined, including all assumptions | Pages 11–13. Non-inferiority calculation using the swCRTdesign package; 37 clusters, 13 periods, an estimated 22,625 episodes (≈47 per cluster-period), α = 0.025 one-sided, ICC 0.009 derived from historical SIR data, baseline 30-day mortality 18%, non-inferiority margin 3 percentage points, power 89%. The justification for the margin, including its expression in absolute terms, is given on pages 11–12. Software: the swCRTdesign package in R. |
| Recruitment | **20** | Strategies for achieving adequate participant enrolment | Page 7 (Recruitment). Clusters were approached via SIR and SFAI. Individual patients are not recruited: all care episodes in participating units are identified through routine SIR registration, which removes the risk of selective enrolment. |
| **Methods: Assignment of interventions** | | | |
| Randomization: sequence generation | **21a** | Who will generate the random allocation sequence and the method used | Pages 15–16 (Randomization Scheme). A constrained randomisation algorithm implemented in R with a fixed random seed, generated by a named investigator; the seed, generating script and equivalent Stata and Python implementations are archived for reproduction. |
|  | **21b** | Type of randomization and details of any factors for stratification | Pages 15–16. Restricted (constrained) allocation of 37 clusters to 10 sequences with 3–4 clusters per sequence, stratified by ICU category (SIR levels 1–3) and ICU size (admissions per year), with one pre-specified constraint linking the two staff-sharing ICUs into a single cluster. |
| Allocation concealment mechanism | **22** | Mechanism used to implement the random allocation sequence, describing any steps to conceal the sequence until interventions are assigned | Page 16 (Randomization Scheme). Explains that because randomisation is at cluster level with no individual patient enrolment there are no enrolling personnel whose behaviour could be influenced by foreknowledge of the next allocation, so conventional allocation concealment does not apply in the individual-participant sense. The generated sequence was held by the trial statistician and not disclosed to participating units before allocation was complete; each cluster was then notified of its own transition date 6–8 weeks in advance. The full allocation list is stored securely with the trial master file. |
| Implementation | **23** | Whether the personnel who will enrol and those who will assign participants to the interventions will have access to the random allocation sequence | Pages 7 and 16. There is no individual patient enrolment: all care episodes in participating units are identified through routine SIR registration, so no enrolling personnel exist who could be influenced by knowledge of the sequence. Page 16 states who generated and who held the allocation sequence and how it is stored. |
| Blinding | **24a** | Who will be blinded after assignment to interventions | Pages 18–19 (Blinding). ICU personnel, treating physicians and local investigators cannot be blinded; patients are not formally blinded; no blinding of registry personnel is claimed. Data management and analysis are conducted with the treatment condition held as a coded exposure variable until the analysis code is finalised, so the analyst is blinded during code development. |
|  | **24b** | If blinded, how blinding will be achieved and description of the similarity of interventions | Not applicable and explained on pages 18–19: the intervention changes unit-level clinical routines, so blinding of clinical staff is impossible and no attempt is made. Protection against detection bias rests instead on objective outcome ascertainment through registry linkage to the population register. |
|  | **24c** | If blinded, circumstances under which unblinding is permissible, and procedure for revealing allocation during the trial | Page 19 (Blinding). Not applicable and explicitly stated: because the trial is open-label at the cluster level there is no individual participant allocation that could be revealed, and no unblinding procedure is required. The analyst's coded exposure variable is revealed only after the statistical analysis code has been finalised and archived. |
| **Methods: Data collection, management, and analysis** | | | |
| Data collection methods | **25a** | Plans for assessment and collection of trial data, including processes to promote data quality and a description of trial instruments | Pages 20–21. Outcome data are extracted from SIR, with the specific variables listed. Process data are collected by designated local clinical monitors on a purpose-built eCRF; monitors receive standardised training before site initiation. Data quality procedures are described on page 21. |
|  | **25b** | Plans to promote participant retention and complete follow-up, including outcome data collected for participants who discontinue or deviate | Page 19 (Blinding, final paragraph), stated explicitly: follow-up for the primary outcome is complete by construction because 30-day mortality is ascertained through SIR's linkage to the Swedish population register rather than through participant contact, so loss to follow-up is not a meaningful mechanism in this trial. Missing data for covariates are addressed on page 18. |
| Data management | **26** | Plans for data entry, coding, security, and storage, including processes to promote data quality | Page 21 (Data Quality and Validation; Data Security). Two-level auditing, cross-checking of eCRF entries against the electronic health record and departmental consumables orders, retraining and correction procedures, secure SIR servers, removal of personal identifiers for analysis datasets, and handling according to Swedish data protection regulation. |
| Statistical methods | **27a** | Statistical methods used to compare groups for primary and secondary outcomes, including harms | Pages 17–18. Generalised linear mixed-effects model with logit link, categorical monthly time effects, pre-specified baseline covariates and a random intercept for cluster; adjusted absolute risk difference obtained by regression standardisation with cluster bootstrap or delta-method confidence intervals; non-inferiority declared if the upper 95% bound lies below 3 percentage points. Time-to-event secondary outcomes use cause-specific Cox models with a gamma frailty and death as a competing event. Harms and the secondary safety outcomes are analysed as described on pages 17–18 and 19. The multiplicity position is stated on page 17. |
|  | **27b** | Definition of who will be included in each analysis, and in which group | Page 17 (intention-to-treat at the cluster level; each care episode analysed under the exposure condition prevailing at admission; patient nested within cluster as a random intercept, with a sensitivity analysis restricted to first admissions) and page 7 (exclusion of episodes beginning within the first hour after transition). |
|  | **27c** | How missing data will be handled in the analysis | Page 18 (Handling of missing data). Complete-case analysis as the primary approach given >95% SIR completeness, multiple imputation by chained equations if missing data exceed 10% for key variables, assessment and reporting of missing data patterns, and a sensitivity comparison of complete-case and imputed results. |
|  | **27d** | Methods for any additional analyses (e.g., subgroup and sensitivity analyses) | Page 18. Sensitivity analyses cover alternative time-trend specifications and correlation structures, outlying clusters, baseline period length, minimal detectable difference, gradual onset of treatment effect via a categorical time-since-switch variable, and a per-protocol analysis if cluster drop-out occurs. The pre-specified subgroup analysis is by SIR ICU category. |
| **Methods: Monitoring** | | | |
| Data monitoring committee | **28a** | Composition of the DMC, its role and reporting structure, independence, conflicts of interest; or an explanation of why a DMC is not needed | Page 19 (Safety Monitoring). A formal DMC with interim efficacy analyses is not established, and the reasons are given. Oversight is provided instead by a named independent safety review group of two senior intensivists who are not trial investigators, with its role and reporting structure described. |
|  | **28b** | Explanation of any interim analyses and stopping guidelines, including who has access to interim results and makes the final decision to terminate | Page 19. No interim efficacy analysis is planned and there is no stopping rule for benefit. Monthly unblinded safety reports are reviewed by the safety review group, with a pre-specified quantitative trigger for review; the group may recommend continuation, modification, suspension at individual ICUs, or termination, with the principal investigator and steering committee responsible for acting on recommendations. |
| Trial monitoring | **29** | Frequency and procedures for monitoring trial conduct; if there is no monitoring, give explanation | Pages 10–11 (three five-day local monitoring periods per site, plus continuous high-resolution EHR extraction where available) and 21 (two-level auditing: SIR's routine registry-wide validation, and trial-specific audits cross-checking eCRF entries against source data, with standardised reporting to the steering committee). |
| **Ethics** | | | |
| Research ethics approval | **30** | Plans for seeking research ethics committee/institutional review board approval | Page 22 (Ethical Considerations, IRB/Regulatory Approval). Approved by the Swedish Ethical Review Authority, Dnr 2025-01558-01, 2025-06782-02 and 2025-07290-02; conducted in accordance with the Declaration of Helsinki. |
| Protocol amendments | **31** | Plans for communicating important protocol modifications to relevant parties | Page 22 (Protocol amendments). Amendments are proposed to and approved by the steering committee; substantive amendments are submitted to the Swedish Ethical Review Authority before implementation, recorded in an updated protocol version with date, change and reason, reflected in the ClinicalTrials.gov record, communicated to all participating units and to the independent safety review group, and reported in the trial publication. |
| Consent or assent | **32a** | Who will obtain informed consent or assent from potential participants or authorized proxies, and how | Page 22 (Consent to participate). Individual consent is waived, with four stated grounds: the registry-based design using existing data collection systems, the quality-improvement nature of the interventions, the minimal-risk profile, and the impracticality of individual consent in the ICU setting. The waiver was approved by the Swedish Ethical Review Authority. |
|  | **32b** | Additional consent provisions for collection and use of participant data and biological specimens in ancillary studies | Page 22 (Consent to participate), stated explicitly: no biological specimens are collected for trial purposes and the trial uses only data already recorded during routine care and registered in SIR. No ancillary studies requiring separate consent provisions are planned; any future ancillary use of trial data would require a separate application to the Swedish Ethical Review Authority. |
| Confidentiality | **33** | How personal information about potential and enrolled participants will be collected, shared, and maintained to protect confidentiality | Page 21 (Data Security). SIR manages secure servers, personal identifiers are removed for analysis datasets, secure transfer protocols are used, and all handling follows Swedish data protection regulation. Care episode IDs are for SIR internal use only. |
| Ancillary and post-trial care | **34** | Provisions, if any, for ancillary and post-trial care, and for compensation to those who suffer harm from trial participation | Pages 19 and 22 (Harms; Risk-Benefit Assessment). No trial-specific post-trial intervention or ancillary-care arrangement is planned, with the reasons given; after the trial each ICU decides locally whether to continue the protocols. Patients are covered throughout by the ordinary Swedish patient-injury insurance (patientförsäkringen, LÖF), and no trial-specific compensation mechanism exists. |

**Citation and licence**

Checklist item descriptions are reproduced from: Chan A-W, Boutron I, Hopewell S, Moher D, Schulz KF, Collins GS, et al. SPIRIT 2025 statement: updated guideline for protocols of randomised trials. BMJ. 2025;389:e081477. doi:10.1136/bmj-2024-081477

© 2025 Chan A-W et al. Open Access under the Creative Commons Attribution licence (CC BY 4.0). It is recommended that this checklist be read in conjunction with the SPIRIT 2025 Explanation and Elaboration document (BMJ 2025;389:e081660). See www.consort-spirit.org
